# Synthesis of MnFe_2_O_4_ Nanoparticles and Subsequent Prussian Blue Functionalization for a Novel Composite Photothermal Material

**DOI:** 10.3390/nano15171382

**Published:** 2025-09-08

**Authors:** Mengyu Wang, Ming Zhang, Zhihan Liang, Min Su

**Affiliations:** 1 School of Chemical Engineering, Hebei University of Technology, Tianjin 300401, China; 2 Xiucun Pharmaceutical Development Co., Ltd., Tianjin 300450, China

**Keywords:** MnFe_2_O_4_ magnetic nanoparticles, particle size regulation, Prussian blue, photothermal performance

## Abstract

MnFe_2_O_4_ magnetic nanoparticles have shown broad application prospects in the field of tumor diagnosis and treatment; however, precise particle size regulation within the 100–200 nm range, as well as the synergistic integration of physical and medical functionalities, remains challenging. As a commonly used method for synthesizing MnFe_2_O_4_ nanoparticles, the solvothermal method has been proven to enable the regulation of the particle size of products, particularly its ability to utilize the viscosity of solvents as a method for particle size regulation. Therefore, this work investigates the influence of the diethylene glycol (DEG) to ethylene glycol (EG) ratio on particle size regulation in solvothermal synthesis of MnFe_2_O_4_ nanoparticles, and constructs MnFe_2_O_4_@PB nanocomposite materials. The results demonstrate that with the DEG ratio increasing from 0 to 80% in a DEG:EG mixed solvent system, the average particle size of MnFe_2_O_4_ nanoparticles can be reduced from 266 nm to 105 nm. The MPB4.5 sample (MnFe_2_O_4_:PB molar ratio = 5:4.5 in the MnFe_2_O_4_@PB nanostructure) exhibits an optimal photothermal heating effect and good photothermal stability, demonstrating potential as a photothermal therapeutic agent. The resultant MnFe_2_O_4_@PB system provides a strategy for precise particle size regulation and functional integration for photothermal therapy of tumors with magnetic targeting potential.

## 1. Introduction

Magnetic nanoparticles have been widely used in biomedical fields due to their unique properties such as tunable magnetic performance and chemical stability [1,2]. Among numerous types of magnetic nanoparticles, MnFe_2_O_4_ has attracted extensive interest from researchers [3] due to its high magnetization [4] and excellent biocompatibility [5], showing great potential in applications such as targeted drug delivery [6] and magnetic resonance imaging [7].

Particle size control is crucial for facilitating the translational application of MnFe_2_O_4_ in clinical diagnosis and treatment [8]. Previous studies have made progress in controlled synthesis of MnFe_2_O_4_ magnetic nanoparticles, and the commonly adopted methods include co-precipitation [9], hydrothermal/solvothermal [10], thermal decomposition [11], and others. For instance, André C. Horta [12] fine-tuned the size of MnFe_2_O_4_ nanoparticles from 23 nm to 3 nm by adjusting the volume ratio of organic base MIPA to inorganic base NaOH in the co-precipitation method, although significant particle agglomeration was observed. Prashant Kumar [13] used PEG-200 surface-modified hydrothermal synthesis to prepare MnFe_2_O_4_ nanoparticles with reduced agglomeration and an average size of approximately 40 nm. In contrast, the solvothermal method is frequently employed to synthesize dispersed nanoparticles due to its ability to achieve a narrow size distribution and precise shape control [14]. Gaoqian Yuan [15] utilized the solvothermal method to obtain MnFe_2_O_4_ nanoparticles with an average size of 274 nm, featuring high sphericity, good dispersibility, and uniform particle size. Beyond the aforementioned approaches, the thermal decomposition method represents another technique for nanoparticle size regulation [16]; however, it is associated with inherent drawbacks, including the tendency for organic solvent residues [17], high energy consumption, and low yield [2]. In sharp contrast, the solvothermal method further exhibits the advantages of easily available raw materials and low cost [18]. However, despite the fact that recent syntheses of MnFe_2_O_4_ nanoparticles have predominantly focused on small sizes, the development of systems within the 100–200 nm range with both low aggregation and precise size control remains an area demanding further exploration.

Photothermal therapy (PTT), a non-invasive cancer treatment modality [19], capitalizes on the deep-tissue-penetration capability of near-infrared (NIR) light to trigger localized and controlled hyperthermia at the tumor site, thereby achieving selective eradication of cancer cells [20]. In specific therapeutic scenarios, PTT exhibits significant application potential for a variety of tumors. For hepatocellular carcinoma with poor efficacy of traditional single therapy, combining photothermal therapy with chemotherapy enables relatively effective treatment [21]. For superficial breast tissue, light penetration to deep tissues makes photothermal therapy a promising alternative or adjuvant option for breast cancer [22]. To enhance nanoparticle delivery to tumors, Patrick et al. [23] showed that ultrasound-guided slow-controlled injection improves nanoparticle delivery efficiency and precision, offering a clinically translatable real-time method for patient-specific drug administration.

Prussian blue (PB) exhibits strong metal-to-metal charge transfer absorption in the near-infrared region [24], and possesses advantages over other photothermal agents (e.g., transition metal chalcogenides) such as low cost, high photostability, and biocompatibility [25], positioning it as a promising photothermal agent. Bingquan Chen et al. [26] proposed a novel PB@Fe-EGCG polyphenol nanolayer nanosystem, which effectively inhibits in vitro cancer cell growth under 808 nm near-infrared laser irradiation. By combining photothermal therapy with chemotherapy, this system achieves a significant inhibition rate of up to 75%. Hwichan Hong [27] synthesized an injectable biocompatible composite of bacterial cellulose and Prussian blue nanoparticles (PB NPs), which exhibits good in vivo retention and enables tumor elimination through repeated photothermal therapy (PTT).

In selecting photothermal agent carriers, superparamagnetic nanoparticles outperform other photothermal carriers such as poly (lactic-co-glycolic acid) (PLGA) [28] as they efficiently deliver nanocomposites to tumors under an external magnetic field [29]. Among these magnetic nanoparticles, MnFe_2_O_4_ contains Mn^2+^ ions (with five unpaired d-electrons) that generate magnetic moments to accelerate longitudinal relaxation (T_1_), thereby endowing the material with a stronger T_1_ relaxation effect [30] and exhibiting bright signals in T_1_-weighted imaging [31]. Integrating the magnetic targeting capability of MnFe_2_O_4_ with the near-infrared responsive performance of photothermal materials to construct a synergistic therapeutic system has emerged as a cutting-edge research direction [32]. Notably, compared with smaller particles, photothermal agents loaded with 100–200 nm magnetic nanoparticles are more easily delivered to tumor regions via magnetic guidance [33,34], enabling more precise targeting and further exerting photothermal effects.

This study aims to synthesize MnFe_2_O_4_ magnetic nanoparticles with tunable sizes (100–200 nm), a narrow size distribution, and minimal agglomeration. By decorating MnFe_2_O_4_ nanoparticles with Prussian blue (PB), we constructed a magnetic–photothermal synergistic therapeutic composite material. This system, featuring both potential magnetic targeting capability and near-infrared photothermal conversion performance, offers a novel and high-potential material for magnetically targeted photothermal therapy of tumors and is expected to provide a new high-performance nanoplatform for precise tumor diagnosis and treatment.

## 2. Materials and Methods

### 2.1. Materials and Reagents

Manganese chloride tetrahydrate (MnCl_2_∙4H_2_O), iron (III) chloride hexahydrate (FeCl_3_∙6H_2_O), anhydrous sodium acetate (CH_3_COONa), polyethylene glycol 2000 (PEG2000), ethylene glycol (EG), diethylene glycol (DEG), sodium ferrocyanide decahydrate (Na_4_Fe(CN)_6_∙10H_2_O), hydrochloric acid (36–38%), and polyvinylpyrrolidone K30 (PVP K30) were obtained from Aladdin Biochemical Technology Co., Ltd., Shanghai, China. All chemical reagents were used directly without further purification.

### 2.2. Synthesis of MnFe_2_O_4_ Nanoparticles

A total of 4.0 mmol of MnCl_2_∙4H_2_O (0.8000 g) and 8.0 mmol of FeCl_3_∙6H_2_O (2.1624 g) were added to 80 mL of solvent (either ethylene glycol or diethylene glycol) and dispersed uniformly by mechanical stirring. Subsequently, 4.0 mmol of CH_3_COONa (5.2499 g) and 3.2586 g of PEG2000 were added to the above-mentioned solution. The mixture was mechanically stirred for 1 h, then transferred into a hydrothermal autoclave (Shanghai Xiniu Laibo Instrument Co., Ltd., Shanghai, China; 100 mL), which was then placed in a 200 °C forced-air drying oven (Tianjin Laibote Rui Instrument Equipment Co., Ltd., Tianjin, China; GFL-45) for a 12 h reaction, followed by cooling to room temperature. The product was washed several times with deionized water and anhydrous ethanol, and then dried in a vacuum oven (Shanghai Lichen Bangxi Instrument Technology Co., Ltd., Shanghai, China; DZF-6050AB) at 60 °C for 24 h for subsequent characterizations.

### 2.3. Synthesis of MnFe_2_O_4_@PB Nanocomposite

In total, 0.5470 g of Na_4_Fe(CN)_6_·10H_2_O and 0.1094 g of PVP were dissolved in 25 mL of hydrochloric acid solution (pH = 2) under magnetic stirring at 400 rpm to form solution A. An amount of 0.6487 g of FeCl_3_∙6H_2_O and 0.1297 g of PVP were dissolved in 25 mL of hydrochloric acid solution (pH = 2). After complete dissolution, 0.6919 g of MnFe_2_O_4_ nanoparticle powder was added to the solution, followed by stirring for an additional 20 min to form solution B.

Solution A was then added dropwise to Solution B under continuous magnetic stirring. After 30 min of reaction, the mixture was washed repeatedly with deionized water and anhydrous ethanol. The product was collected by centrifugation at 2000 rpm and dried under vacuum at 60 °C for 24 h.

Prussian-blue-coated MnFe_2_O_4_ magnetic nanoparticles (MnFe_2_O_4_@PB) were synthesized using the procedure described above. By varying the molar amounts of Na_4_Fe(CN)_6_∙10H_2_O (1.8 mmol, 3.6 mmol) and FeCl_3_∙6H_2_O (2.4 mmol, 4.8 mmol), nanoparticles with different MnFe_2_O_4_:PB molar ratios (5:1, 5:2, 5:3, 5:4, 5:4.5, 5:5.4) were achieved, labeled as MPB1, MPB2, MPB3, MPB4, MPB4.5, and MPB5.4, respectively (Table 1).

### 2.4. Characterization

The products were characterized by powder X-ray diffraction using a D8 FOCUS X-ray diffractometer from Bruker AXS GmbH, Germany. The microstructure of the prepared MnFe_2_O_4_ materials was observed via a Quanta 450 FEG scanning electron microscope (SEM) from FEI Hong Kong Limited. Infrared (IR) spectroscopy tests on the samples were conducted using an IRAffinity-1S infrared spectrometer from Shimadzu Corporation, Japan. The hysteresis loop measurements were performed using a LakeShore 7404 magnetometer from LakeShore Cryotronics, Inc., Westerville, OH, USA. The UV–Vis–NIR spectra were measured using a UV2700 UV–Vis spectrophotometer from Shimadzu Corporation, Kyoto, Japan.

For the photothermal performance experiments, a VCL-MO-2W laser from Beijing Honglan Optoelectronic Technology Co., Ltd., Beijing, China was used to irradiate the samples with a wavelength of 808 nm and an emission power density of 3.41 W/cm^2^. Thermal infrared images were captured, and temperature measurements were recorded using an S65 infrared thermal imager from FLIR Systems, Wilsonville, Oregon, USA.

## 3. Results and Discussion

### 3.1. Characterizations of MnFe_2_O_4_ Nanoparticles

**Figure 1:**
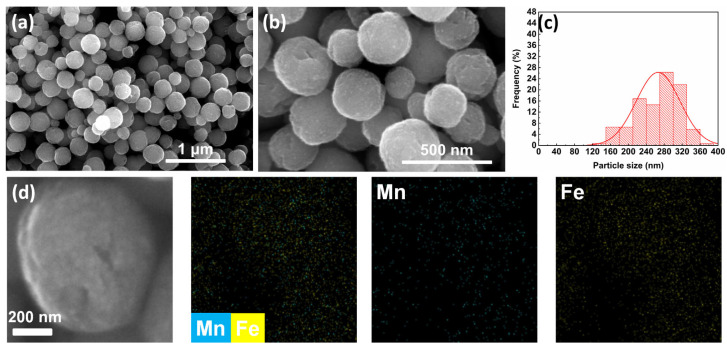
(**a**) SEM image at 100,000× magnification, scale bar = 1 μm; (**b**) SEM image at 300,000× magnification, scale bar = 500 nm; (**c**) particle size distribution diagram; (**d**) SEM elemental mapping images of MnFe_2_O_4_ synthesized using ethylene glycol as solvent.

In the synthesis of MnFe_2_O_4_ nanoparticles, ethylene glycol (EG) is commonly used as a solvent in solvothermal methods due to its strong coordination ability with metal ions—which forms stable chelates—and its high viscosity that can limit the reaction rate [35] These properties enable EG to form nanoparticles with uniform morphology and prevent agglomeration in the synthesis of magnetic nanoparticles [36]. In this work, EG was first employed as the primary solvent for synthesis. The morphology of the synthesized MnFe_2_O_4_ sample was observed by SEM, and the particle size distribution diagram was plotted based on the statistical data obtained from the SEM images. As shown in Figure 1, the average particle size of the MnFe_2_O_4_ sample was 266.37 ± 48.83 nm with a relative standard deviation (RSD) of 18.33%. The SEM elemental mapping shows that Mn and Fe elements exhibit a homogeneous distribution in the MnFe_2_O_4_ sample, with their distribution regions overlapping with one another. The morphology exhibited a dispersed spherical state, although the particle size exceeded the target range of 100–200 nm.

In the solvothermal process, when the energy barrier between nanoscale precursors is eliminated, a limited number of “agglomeration centers” are rapidly formed, and these precursors subsequently grow rapidly at the expense of consuming surrounding constituent subunits [37]. Based on this theory, the existing experimental reaction process may have the problem that the excessively fast growth rate leads to large particle size and broad particle size distribution of MnFe_2_O_4_. Combined with the literature research [38], it is found that at a pressure of 0.098 MPa, the viscosity of diethylene glycol (DEG) is always higher than that of ethylene glycol (EG)—at 293.15 K, the viscosity of DEG is 36.52 mPa∙s and that of EG is 21.10 mPa∙s; at 464.40 K, the viscosity of DEG is 0.789 mPa∙s and that of EG is 0.652 mPa∙s. In addition, this viscosity difference between solvents may have an impact on the solvothermal synthesis process of nanoparticles [39]. Therefore, DEG was selected as a secondary solvent here to investigate the possible influence of different solvent systems on size-controlled synthesis of MnFe_2_O_4_ nanoparticles. The experiment was designed with different ratios of EG to DEG, ranging the proportion of DEG in the solvent system from 50% to 80%. The particle sizes of different groups of experiments are shown in Table 2.

**Figure 2:**
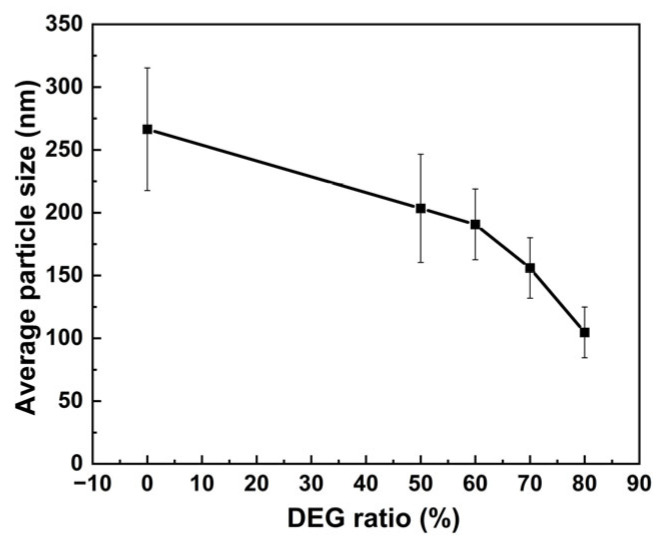
Variation in the average particle size of MnFe_2_O_4_ with the ratio of diethylene glycol.

These data are further summarized as a trend in Figure 2, which clearly illustrates the variation in particle sizes of solvothermally synthesized MnFe_2_O_4_ nanoparticles with diethylene glycol ratios. With the increase in the proportion of diethylene glycol in the system, the average particle size of MnFe_2_O_4_ nanoparticles gradually decreased from 266 nm to 105 nm.

**Figure 3:**
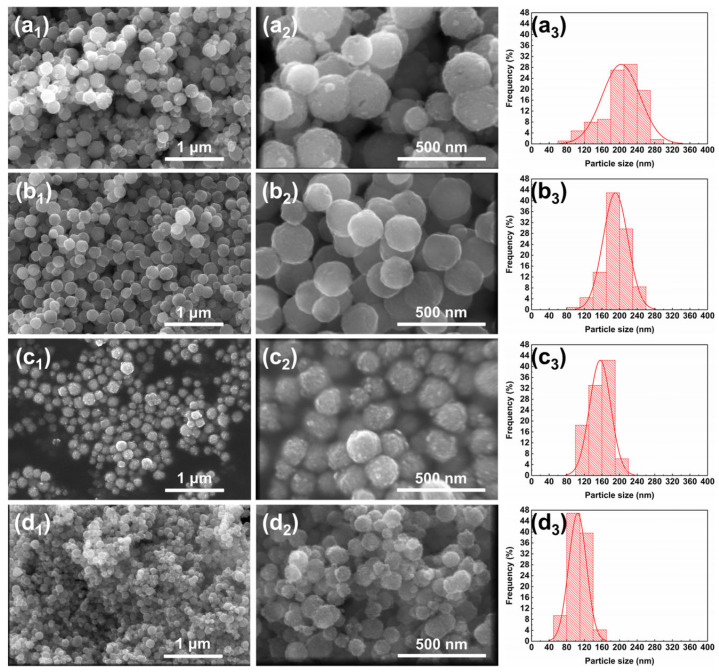
SEM images and particle size distribution of MnFe_2_O_4_ nanoparticles synthesized with different DEG ratios: (**a_1_**–**a_3_**) 50%; (**b_1_**–**b_3_**) 60%; (**c_1_**–**c_3_**) 70%; (**d_1_**–**d_3_**) 80%. For each ratio, a_1_/b_1_/c_1_/d_1_ show low-magnification (100,000×) SEM images, scale bar = 1 μm; a_2_/b_2_/c_2_/d_2_ show high-magnification (300,000×) SEM images, scale bar = 500 nm; a_3_/b_3_/c_3_/d_3_ show the corresponding particle size distribution diagrams.

The SEM images and particle size distribution diagrams of the samples are shown in Figure 3a_1_–a_3_, depicting the solvent system with 50% diethylene glycol (DEG), where the average particle size of MnFe_2_O_4_ nanoparticles is 203.43 ± 43.05 nm. Compared to the ethylene glycol system, the particle size significantly decreases, approaching the target size of 200 nm. However, the relative standard deviation (RSD) increases slightly to 21.16%. When the DEG proportion further increases to 60% [Figure 3b_1_–b_3_], the average particle size of MnFe_2_O_4_ is 190.69 ± 28.18 nm with an RSD of 14.78%, representing the smallest RSD among the designed proportional conditions. This sample shows the narrowest particle size distribution and the most uniform particle growth. In Figure 3c_1_–c_3_, with a DEG proportion of 70%, the average particle size is 155.99 ± 24.06 nm, showing a substantial decrease from the previous sample, while the RSD remains relatively stable at 15.42%. For Figure 3d_1_–d_3_ with 80% DEG, the average particle size of MnFe_2_O_4_ is 104.69 ± 20.19 nm, and the RSD increases to 19.29%, accompanied by a broadened particle size distribution and slight aggregation between particles.

During the process of increasing the DEG proportion from 0% to 80%, the particle size of MnFe_2_O_4_ nanoparticles decreases from approximately 266 nm to 105 nm, basically achieving the target size control within 100–200 nm. Overall, the MnFe_2_O_4_ nanoparticles exhibit regular spherical morphologies with minimal aggregation. The realization of such particle size regulation effect can be attributed to the higher viscosity of diethylene glycol (DEG) and its stronger coordination ability with metal ions compared with ethylene glycol (EG) [40]. When pure EG is used, the crystal nuclei agglomerate rapidly due to their high surface energy and the existence of magnetic dipole–dipole interactions, eventually yielding nanoparticles with relatively large particle sizes [37]. After the introduction of diethylene glycol (DEG), compared with ethylene glycol (EG), the bulkier DEG can form more stable coordination complexes with metal ions. This not only slows down the growth rate of MnFe_2_O_4_ grains but also is more conducive to increasing the number of “agglomeration centers” [41]. Additionally, due to the higher viscosity of the system, MnFe_2_O_4_ crystal nuclei cannot agglomerate into larger spheres [42]; ultimately, MnFe_2_O_4_ with a smaller particle size is obtained.

From the perspective of particle size distribution, the relative standard deviation (RSD) of the size distribution for the samples in this study is approximately 15% or higher, which is slightly higher than that of MnFe_2_O_4_ synthesized via the solvothermal method (with a particle size of 274 ± 40 nm and a corresponding RSD of 14.6%) as previously reported by Gaoqian Yuan [15]. Overall, this result indicates that there are slight limitations in the monodispersity of the samples in this study. The particle size distribution can be further narrowed by optimizing synthetic conditions with such strategies as screening and using alternative surfactants [43].

**Figure 4:**
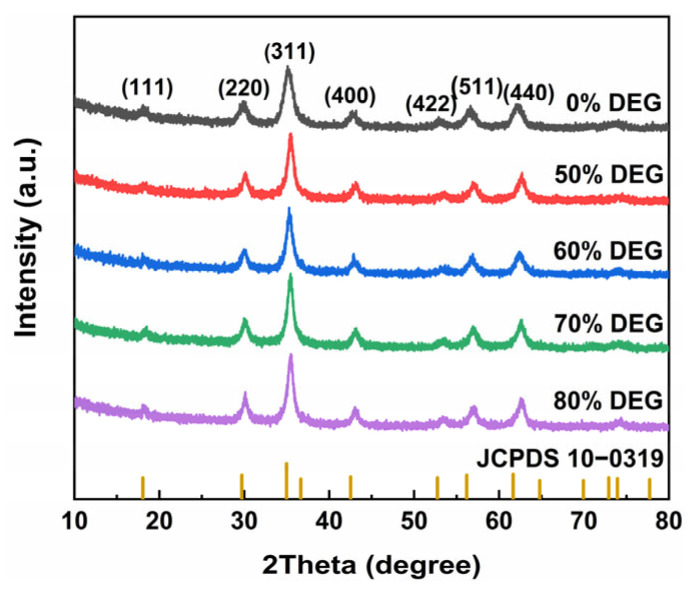
XRD pattern of MnFe_2_O_4_ nanoparticles samples synthesized with different DEG:EG ratios.

The crystal structure of the MnFe_2_O_4_ nanoparticles was determined by XRD, and the corresponding XRD patterns are shown in Figure 4. Characteristic diffraction peaks were observed at 2θ = 18.18°, 29.90°, 35.20°, 42.71°, 53.05°, 57.08°, and 62.20°, corresponding to the (111), (220), (311), (400), (422), (511), and (440) crystal planes of the MnFe_2_O_4_ spinel structure (JCPDS 10-1039), indicating successful synthesis of MnFe_2_O_4_ nanoparticles via the solvothermal method. The XRD patterns of samples synthesized with varying diethylene glycol ratios in the solvent system were all in good agreement with the standard card, confirming that the nanoparticles synthesized with different solvent systems remained as MnFe_2_O_4_.

### 3.2. Characterizations of MnFe_2_O_4_@PB Nanocomposite

**Figure 5:**
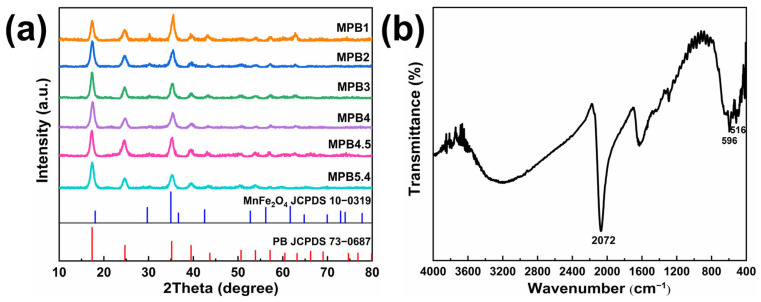
(**a**) XRD pattern, MPB1, MPB2, MPB3, MPB4, MPB4.5, and MPB5.4 represent different MnFe_2_O_4_:PB molar ratios (5:1, 5:2, 5:3, 5:4, 5:4.5, 5:5.4), respectively; (**b**) infrared spectrum of MnFe_2_O_4_@PB nanocomposite.

In the synthesis of MnFe_2_O_4_@PB nanostructure, when the solution containing Na_4_[Fe(CN)_6_]∙10H_2_O is mixed with a solution of FeCl_3_∙6H_2_O, the mixed solution rapidly develops a bright blue color, indicating the formation of Prussian blue (PB). The powder diffraction pattern of the MnFe_2_O_4_@PB sample was first characterized, as shown in Figure 5a. For instance, sample MPB1 showed its peak positions at 30.22°, 35.53°, and 42.13°, matching the MnFe_2_O_4_ JCPDS standard (Card No.: 10-0319), which corresponds to the crystal planes (220), (311), and (400), respectively. Compared to pure MnFe_2_O_4_ nanoparticles, the additional diffraction peaks at 2θ = 17.35°, 24.80°, and 39.55° belong to the (200), (220), and (420) crystal planes of the Prussian blue standard card (73-0687) [44], confirming the combination of Prussian blue and MnFe_2_O_4_ materials.

The sample was further analyzed by infrared (IR) spectroscopy, as shown in Figure 5b. The peaks at 516 cm^−1^ and 596 cm^−1^ are ascribed to the stretching vibrations of Mn-O and Fe-O bonds in MnFe_2_O_4_, respectively [45]. The broad peaks at 1630 cm^−1^ and 3121 cm^−1^ are attributed to the O-H bending vibration of adsorbed water molecules and the stretching vibration of -OH groups [46]. A strong characteristic peak at 2072 cm^−1^ originates from the C≡N stretching vibration in the Fe^2+^-CN-Fe^3+^ chemical unit of Prussian blue [47], further verifying the combination of Prussian blue and MnFe_2_O_4_.

**Figure 6:**
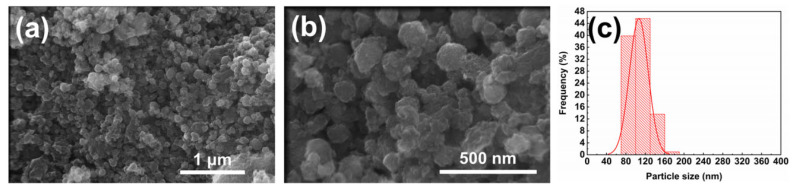
(**a**) SEM image at 100,000× magnification, scale bar = 1 μm; (**b**) SEM image at 300,000× magnification, scale bar = 500 nm; (**c**) particle size distribution diagram of MnFe_2_O_4_@PB nanocomposite.

The SEM images and particle size distribution in Figure 6 show that the PB-coated sample maintains a near-spherical particle morphology with an average particle size of 106.66 ± 19.45 nm and an RSD of 18.24%. The particle size and distribution exhibit no significant changes before and after coating, remaining within the 100–200 nm range.

**Figure 7:**
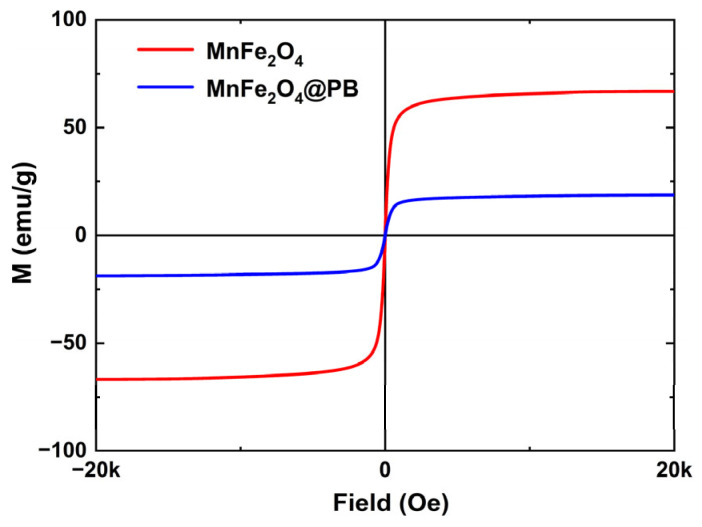
Magnetization hysteresis loops of MnFe_2_O_4_ and MnFe_2_O_4_@PB.

To verify the magnetic properties of the synthesized samples and their potential for future magnetic targeting applications, their magnetic properties were measured using a vibrating sample magnetometer (VSM), and the resulting hysteresis loops are presented in Figure 7. Both MnFe_2_O_4_ and MnFe_2_O_4_@PB exhibit small coercivity and remanence, indicating that both possess excellent superparamagnetic properties [48]. The saturation magnetization (*M*_s_) of MnFe_2_O_4_ is 66.53 emu/g, while that of MnFe_2_O_4_@PB is 18.74 emu/g. Specifically, the saturation magnetization (*M*_s_) of MnFe_2_O_4_ in this study is essentially consistent with the upper limit of the 45–67 emu/g range for samples prepared by the chemical oxidation method [49], and it is also significantly higher than the 29.3 emu/g of the samples reported by Li et al. [50]. After modification with Prussian blue, MnFe_2_O_4_ still retains considerable magnetic ability, with its saturation magnetization (*M*_s_) being higher than that of the multifunctional nanoparticles (MFNPs) containing Fe_3_O_4_ and PB components reported by Du et al. (approximately 8 emu/g) [51]. Notably, Du’s study has confirmed that their prepared MFNPs, under the guidance of an external magnetic field, can achieve a tumor accumulation level twice as high as that in the group without a magnetic field, and the photothermal therapeutic efficacy can be enhanced through magnetic targeting. Therefore, the MnFe_2_O_4_@PB nanocomposites in this study, which possess a higher saturation magnetization (18.74 emu/g), are expected to exhibit stronger magnetic response ability. This magnetic response characteristic enables it to achieve directional movement and enrichment under the guidance of an external magnetic field, demonstrating good magnetic targeting potential and providing an important basis for further exploration of its combined application with photothermal performance in future research.

### 3.3. The Photothermal Properties of MnFe_2_O_4_@PB Nanocomposite

**Figure 8:**
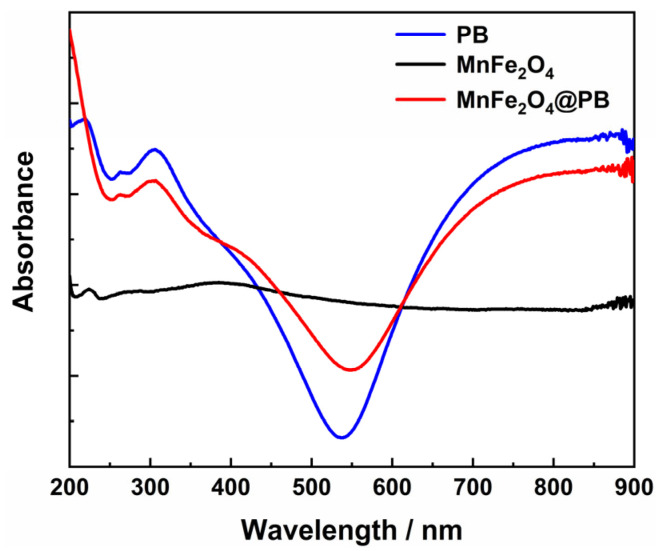
UV–Vis–NIR spectra of PB, MnFe_2_O_4_, and MnFe_2_O_4_@PB.

The UV–Vis–NIR spectrum of the obtained MnFe_2_O_4_@PB, as shown in Figure 8, shows that it has a strong absorption capacity for near-infrared light (650–900 nm), which is basically consistent with the near-infrared absorption of Prussian blue alone. This indicates that the Prussian blue modification strategy endows MnFe_2_O_4_ with the characteristic of strong near-infrared absorption, providing crucial optical property support for expanding the near-infrared photothermal performance of MnFe_2_O_4_-based materials. Therefore, this study selected an 808 nm near-infrared laser, consistent with numerous previous studies [52,53], to test the photothermal performance of the samples.

**Figure 9:**
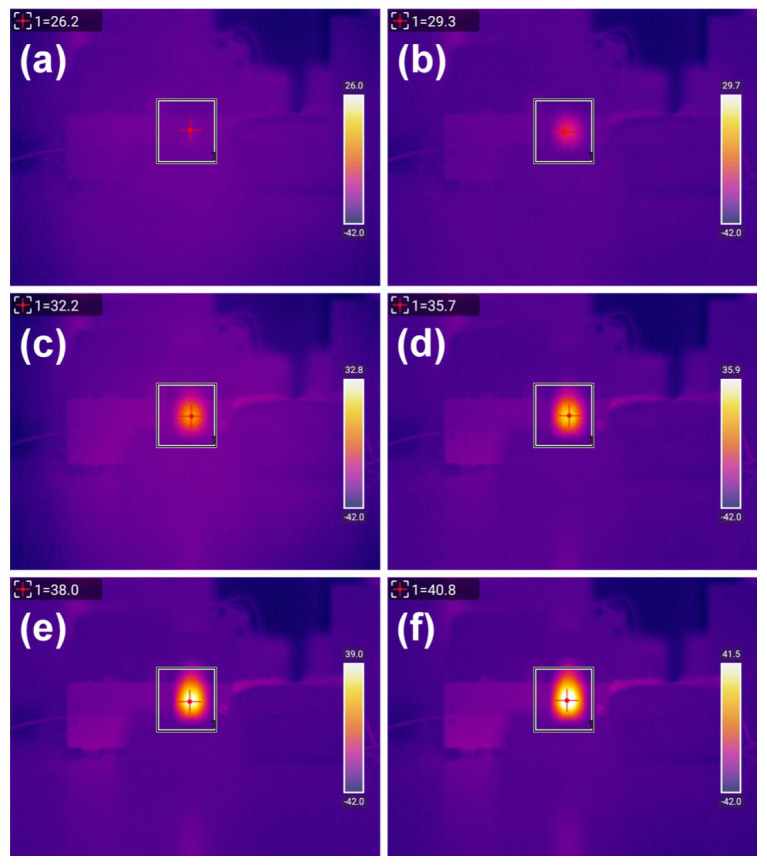
Infrared thermal images of the MPB1 sample under laser irradiation at different times: (**a**) 0 min, (**b**) 1 min, (**c**) 2 min, (**d**) 3 min, (**e**) 4 min, (**f**) 5 min. Particle concentration: 2 g/L; number of repetitions: 3 times; laser wavelength: 808 nm; laser power density: 3.41 W/cm^2^.

Photothermal heating experiments were conducted on six groups of MnFe_2_O_4_@PB samples with different MnFe_2_O_4_:PB molar ratios and a control group of water, under the following experimental conditions: particle concentration of 2 g/L; number of repetitions, 3 times; laser wavelength of 808 nm; and laser power density of 3.41 W/cm^2^. An infrared thermal imager was used to record the temperature during the experiments. Taking sample MPB1 for instance, the infrared thermal images showing the temperature changes during the experiment—presented in Figure 9a–f sequentially—depict the temperature distribution of MPB1 under laser irradiation at 0, 1, 2, 3, 4, and 5 min. As the irradiation time increased, the color of the sample area in the images gradually shifted from red to brighter hues, indicating a continuous increase in temperature.

The photothermal heating experiments under laser irradiation were repeated three times for samples with different modification ratios in each group. The heating data are shown in Figure 9; this displays the infrared thermal images of the MPB1 sample under laser irradiation at different times: (a) 0 min, (b) 1 min, (c) 2 min, (d) 3 min, (e) 4 min, and (f) 5 min. The conditions applied were as follows: particle concentration: 2 g/L; number of repetitions: 3 times; laser wavelength: 808 nm; laser power density: 3.41 W/cm^2^.

**Figure 10:**
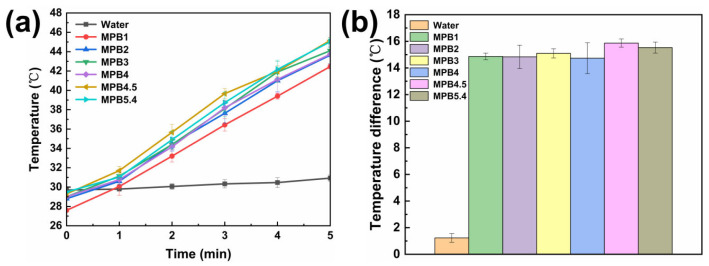
(**a**) Temperature increase over time of various samples under laser irradiation; (**b**) temperature difference data of various samples between the 5th minute of laser irradiation and before illumination. The sample size (*n* value) = 3. The points in the figure represent the mean values, and the error bars indicate the standard deviation (SD).

Table 3, and the corresponding heating data plot is illustrated in Figure 10a. It can be observed that for the control group containing only water, the temperature curve is extremely smooth. After laser irradiation, the temperature increase is at most 1.6 °C. In contrast, the experimental groups with MnFe_2_O_4_@PB samples exhibit significant and stable temperature increases after laser irradiation.

During the 5 min photothermal heating test, sample MPB1 shows the smallest temperature increase, with an increment of only 14.6 °C. This is presumably because of a small amount of Prussian blue coated on the surface of MnFe_2_O_4_ nanoparticles, resulting in a limited charge transfer of Prussian blue upon laser irradiation and, thus, less heat generation. Sample MPB4.5 exhibited the highest temperature increase, reaching a maximum of 45.5 °C, which falls within the medical temperature range of 42–46 °C for triggering apoptotic programs to treat cancer cells [54]. However, for sample MPB5.4, with a further increased modification ratio, the heating effect is inferior to that of MPB4.5, with a maximum temperature increment of 16 °C. It is speculated that as the modification ratio of MnFe_2_O_4_ to PB reaches 5:4.5, Prussian blue has achieved the maximum coating on MnFe_2_O_4_ nanoparticles under the current experimental conditions. For MPB5.4, the excessive content of Prussian blue in the experiment may cause aggregation during the coating process, thereby affecting the photothermal heating performance.

Figure 10b presents a data plot of the temperature differences between the 5th minute of heating and the initial temperature before light irradiation for each group of samples. This plot clearly illustrates the comparison of the 5 min photothermal heating effects among six groups of samples with different molar modification ratios and the control group. Among these samples, MPB4.5 exhibits the greatest temperature increase, indicating that the sample under the condition of MPB4.5 has stronger heating capacity upon near-infrared laser irradiation and a better photothermal effect.

To verify the statistical significance of the differences between groups, a one-way analysis of variance (ANOVA) was performed on the photothermal heating data of six groups of samples with different molar modification ratios (*n* = 3 for each group). The results showed that the difference between groups was significant (F = 13.53, P = 1.4 × 10^−4^ < 0.05), indicating that the influence of sample conditions on the photothermal effect was not a random error. Based on the relatively higher heating trend of the MPB4.5 sample, in subsequent studies, investigation and analysis of the photothermal stability and photothermal conversion efficiency of samples will focus on the MPB4.5 sample under these conditions.

**Figure 11:**
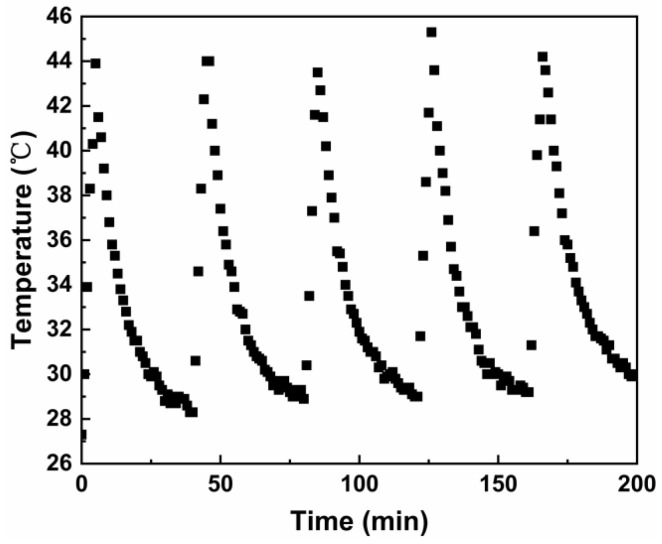
Heating and natural cooling cycle of MPB4.5 under laser irradiation.

Sample MPB4.5 was prepared as a 2 g/L test solution for the examination of photothermal stability, as shown in Figure 11. After undergoing five cycles of stability testing, the solution was still able to reach the maximum temperature (43.9 °C) achieved during the first 5 min of light irradiation, demonstrating that this sample exhibits excellent photothermal stability.

**Figure 12:**
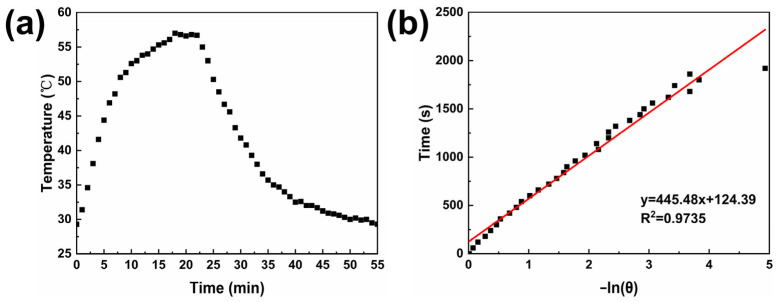
(**a**) Time–temperature relationship of sample MPB4.5 cooling to room temperature after laser irradiation ceases to increase the temperature; (**b**) linear relationship between the negative natural logarithm of the cooling driving force −ln(θ) and time, y = 445.48 x + 124.39, R^2^ = 0.9735.

The photothermal conversion efficiency is a crucial parameter for evaluating the photothermal therapeutic efficacy of photothermal agents [55]. A high photothermal conversion efficiency enables more effective conversion of light energy into heat energy, enhancing the therapeutic effect while minimizing thermal damage to normal tissues. The photothermal conversion efficiency of sample MPB4.5 was investigated using the following method. The sample solution was irradiated with a laser until the temperature ceased to increase, after which the laser was removed, allowing the solution to cool naturally to room temperature. Figure 12a shows the photothermal conversion test curve of sample MPB4.5. In the initial stage of the test, the sample temperature increased rapidly. After approximately 10 min, the rate of temperature increment began to slow down. When the laser irradiation reached 18 min, the solution temperature essentially stopped increasing and remained around 57 °C. Subsequently, the laser was turned off, and the temperature decreased to room temperature.

The photothermal conversion efficacy was calculated according to the following method [56]:

(1) $$\eta =\frac{hA\left(∆T_{max,mix}-∆T_{max,H_{2}O}\right)}{I\left(1-10^{-A_{\lambda }}\right)}$$

(2) $$t=-\frac{m_{d}C_{d}}{hA}ln\left(\theta \right)$$

(3) $$\theta =\frac{∆T}{∆T_{mix,max}}$$

where η corresponds to the photothermal conversion efficiency; h denotes the heat transfer coefficient; A stands for the surface area of the container employed to hold the sample during the light irradiation experiment; ∆T_max,mix_ represents the maximum temperature difference between the sample’s equilibrium temperature and its initial temperature; ∆T_max,H_2_O_ signifies the maximum temperature variation in the aqueous solution from its initial temperature; the mass of water is denoted by m_d_; C_d_ refers to the specific heat capacity of water; ΔT is the temperature difference from the initial temperature in the cooling phase; I denotes the laser power density; and A_λ_ corresponds to the sample’s absorbance at a wavelength of 808 nm.

Given that ∆T_max,mix_ = 27.7 °C, ∆T_max,H_2_O_ = 1.23 °C, m_d_ = 3 g, C_d_ = 4.2 J/(g·°C), I = 3.41 W/cm^2^, and A_λ_= 2.6, by plotting the linear relationship between t and −ln(θ), as shown in Figure 12b, the slope was determined to be 445.48. Substituting this into Equation (2) yields hA = 0.0283. Overall, η was calculated to be 22.02%, with this photothermal conversion efficiency ranking at a moderate level in the field of Prussian blue photothermal applications. This value is close to the photothermal conversion efficiency of PB@Fe-EGCG-HA (23.5%) synthesized by Bingquan Chen [26], lower than the PEGylated PB NPs (36.7%) fabricated by Huajian Chen [57] and exceeds that of Fe_3_O_4_@GdPB NPs (16.1%) described earlier by Kale [58], thus indicating its promising potential as a photothermal therapeutic agent in the near-infrared region.

In the further expansion of the material’s application potential, the development of its imaging function deserves focused attention. MnFe_2_O_4_ magnetic nanoparticles have been actively investigated as MRI contrast agents in diagnostic imaging by other researchers [59]. For example, Mauro Comes Franchini et al. reported MnFe_2_O_4_@SiO_2_@GNRs@PMs for magnetic–photoacoustic–optical trimodal imaging and verified their efficacy and applicability as diagnostic tools in medical research [60]. This points the MnFe_2_O_4_@PB in this paper toward MRI visibility examinations, and future work can further investigate its ability to identify tumor locations and evaluate therapeutic effects. Meanwhile, it should be noted that MRI may perform poorly in quantifying high concentrations of magnetic particles due to signal loss saturation. To overcome this limitation, a radiolabeling strategy compatible with magnetic nanoparticles can be adopted to achieve a more comprehensive evaluation of delivery status [61]. Building on this, imaging-based photothermal therapy enables more precise targeting of lesions. In subsequent preclinical applications, although Prussian-blue-based materials may have potential side effects [62], these effects can be regulated through corresponding strategies, thereby reducing their impact to a great extent [25].

In addition, the solvothermal method and co-precipitation method involved in this study are widely recognized and easily scalable for the synthesis of nanoparticles [63]. As shown in Wang’s research [64], with sodium citrate used as a modifier in the EG-DEG binary solvent, after scaling up the solvothermal reaction system for Fe_3_O_4_@SiO_2_ nanoparticles from 50 mL to 600 mL, only slight changes were observed in the size (approximately 120 nm) and size distribution of the product. This not only provides a robust and scalable approach to increase the yield from the milligram-to-gram scale and confirms the great potential of the solvothermal method for high-yield industrial production but also offers a reference basis for the feasibility of large-scale production of the MnFe_2_O_4_@PB nanocomposite in this study.

## 4. Conclusions

In summary, following the design concept of “precise particle size regulation–functional material integration–photothermal synergistic application”, this study innovatively adjusted the ratio of EG to DEG in the solvothermal method, successfully regulating the average particle size of MnFe_2_O_4_ nanoparticles from 266 nm to 105 nm and providing an effective control strategy for the precise regulation of MnFe_2_O_4_ particle size. By integrating the magnetic targeting property of MnFe_2_O_4_ and the photothermal property of PB, MnFe_2_O_4_@PB nanocomposite photothermal material with magnetic targeting potential was prepared. Among them, the MPB4.5 sample exhibits the strongest photothermal heating effect, good photothermal stability, and a moderate level of photothermal conversion efficiency, which not only confirms the application potential of the MnFe_2_O_4_@PB-based nanoplatform in photothermal therapy but also lays a materials science foundation for its further extension to the field of theranostics.

## Figures and Tables

**Table 1:** Sample designations for MnFe_2_O_4_@PB composites with varying modification molar ratios.

MnFe_2_O_4_:PB	5:1	5:2	5:3	5:4	5:4.5	5:5.4
Sample Name	MPB1	MPB2	MPB3	MPB4	MPB4.5	MPB5.4

**Table 2:** Particle size results of MnFe_2_O_4_ synthesized with different DEG ratios.

DEG Ratio (%)	Average Particle Size (nm)	RSD (%)
0	266.37 ± 48.83	18.33
50	203.43 ± 43.05	21.16
60	190.69 ± 28.18	14.78
70	155.99 ± 24.06	15.42
80	104.69 ± 20.19	19.29

**Table 3:** Photothermal heating data of various MnFe_2_O_4_@PB samples.

Sample	Initial Temperature (°C)	Temperature at 5 min of Light Irradiation (°C)	Temperature Difference (°C)
Water	29.70	30.93	1.23
MPB1	27.60	42.47	14.87
MPB2	28.80	43.63	14.83
MPB3	29.00	44.10	15.10
MPB4	29.00	43.73	14.73
MPB4.5	29.30	45.17	15.87
MPB5.4	29.50	45.03	15.53

Note: particle concentration: 2 g/L; number of repetitions: 3 times; laser wavelength: 808 nm; laser power density: 3.41 W/cm^2^.

## Data Availability

The raw data supporting the conclusions of this article will be made available by the authors on request.
